# Low Dosing Norepinephrine Effects on Cerebral Oxygenation and Perfusion During Pediatric Shock

**DOI:** 10.3389/fped.2022.898444

**Published:** 2022-07-06

**Authors:** Meryl Vedrenne-Cloquet, Judith Chareyre, Pierre-Louis Léger, Mathieu Genuini, Sylvain Renolleau, Mehdi Oualha

**Affiliations:** ^1^Pediatric Intensive Care Unit, AP-HP CHU Necker-Enfants Malades, Paris, France; ^2^Pediatric Intensive Care Unit, AP-HP CHU Trousseau-La Roche Guyon, Paris, France; ^3^Pediatric Intensive Care Unit, AP-HP CHU Robert Debré, Paris, France; ^4^Pediatric Intensive Care Transport Unit, AP-HP CHU Robert Debré, Paris, France

**Keywords:** norepinephrine, pediatric intensive care unit, cerebral perfusion, near infrared spectroscopy, cerebral oxygenation

## Abstract

**Background:**

Cerebral hypoperfusion and impaired oxygen delivery during pediatric critical illness may result in acute neurologic injury with subsequent long-term effects on neurodevelopmental outcome. Yet, the impact of norepinephrine on cerebral hemodynamics is unknown in children with shock. We aimed to describe the norepinephrine effects on cerebral perfusion and oxygenation during pediatric shock.

**Patients and Methods:**

We conducted an observational multicentre prospective study in 3 French pediatric intensive care units. Children <18 years of age excluding traumatic brain injury were included in the study if they need norepinephrine for shock. Systemic and cerebral hemodynamics were compared between the time of initiation of norepinephrine (T_0_), and the steady-state (T_ss_). Cardiac output (CO) was measured using ultrasound. Cerebral perfusion was assessed on middle cerebral arteries (MCA) using transcranial doppler ultrasound. Cerebral tissue oxygen saturation (rScO_2_) was recorded using near infrared spectroscopy, and we calculated cerebral fractional tissue oxygen extraction (cFTOE = SpO_2_-rScO_2_/SpO_2_).

**Main Results:**

Fourteen children (median [IQR] age of 3.5[1; 13.5] years) were included. Norepinephrine at 0.2[0.1; 0.32] μg/kg/min significantly increased mean arterial blood pressure (61[56; 73] mmHg at T_ss_ vs. 49[42;54] mmHg at T_0_, p=10^−3^) without change of CO. MCA velocities, pulsatility index, rScO_2_, and cFTOE did not significantly change between T_0_ and T_ss_. Some individuals observed variations in estimated CBF, which slightly improved in 7 patients, remained unchanged in 5, and was impaired in 2. No patient experienced significant variations of rScO_2_.

**Conclusions:**

Low-dosing norepinephrine, despite a homogeneous and significant increase in arterial blood pressure, had little effects on cerebral perfusion and oxygenation during pediatric shock. This reinforces the need for personalized tailored therapies in this population.

**Trial Registration:**

Clinicaltrials.gov, NCT 03731104. Registered 6 November, 2018. https://clinicaltrials.gov/ct2/show/NCT03731104.

## Introduction

Since the establishment of targeted guidelines for the management of septic shock, patients' survival has dramatically improved (1). However, the failure or delay to restore systemic hemodynamics during early resuscitation of shock impairs outcome. This may be partly due to a persistent regional tissue hypoperfusion that contributes to organ failure (2, 3). Similarly, brain hypoperfusion during shock might lead to microcirculatory dysfunction in the brain, which increases the risk of structural lesions and long-term outcome (4). In adults, cerebral perfusion assessed by transcranial doppler ultrasound (TCD) is impaired during early sepsis (5), and cerebral oxygenation assessed by near-infrared spectroscopy (NIRS) is inversely correlated with blood lactate (6). Norepinephrine is used in patients with shock, especially during sepsis, to restore systemic and regional circulation, but may be ineffective (7, 8). In addition, norepinephrine, while increasing mean arterial pressure (MAP), has been shown to restore cerebral blood flow (CBF), when estimated using TCD not NIRS (9). Critically ill children without previous neurologic dysfunction are exposed to acute brain injury and subsequent neurodevelopmental challenges that may be attributed to many factors including poor oxygen delivery (10–12), while norepinephrine hemodynamic effects are highly variable and unpredictable in this population (13).

Yet, to date, the impact of norepinephrine on cerebral perfusion and oxygenation is unknown in children with shock. TCD reflects cerebral blood flow velocities which may be considered a surrogate for cerebral perfusion in stable conditions (14). Cerebral perfusion monitoring using TCD has already shown its efficacy to detect ischemic injury in several populations of critically ill children (14–16). NIRS offers a continuous and non-invasive cerebral saturation monitoring (17) that might reflect cerebral oxygenation (18), and is routinely used during pediatric cardiac surgery (19).

We aimed to describe the changes of cerebral oxygenation (using NIRS) and perfusion (using TCD) during norepinephrine infusion in critically ill children with shock.

## Methods

### Study Design

This prospective observational study (ClinicalTrials Identifier NCT03731104) was conducted in 3 pediatric intensive care units in Paris, France. The study was allowed by a national institutional review board (South-East IV, ref 2018-A01392-53) before the first inclusion. Oral informed consent was obtained from the parents or legal guardians of the included children. The principal study was planned to describe cerebral circulation in children with hemodynamic failure requiring all types of vasoactive or inotropic treatments. Herein we report our results on patients with norepinephrine only.

### Population

Children <18 years old were included if they needed norepinephrine for shock defined by inadequate tissue perfusion with or without arterial hypotension (20, 21). Clinical signs of inadequate tissue perfusion included any of the following: decreased or altered mental status; prolonged (>2 s) or flash capillary refill (defining, respectively, cold and warm shock), mottled cool extremities; diminished pulses or bounding peripheral pulses and wide pulse pressure; decreased urine output (<1 mL/kg/h) (22). Hypotension was defined according to systolic and mean blood pressure depending on the patient's age (21) since the restoration of a normal mean arterial pressure constitutes one of the most important targets during shock resuscitation (20) Patients were excluded in case of traumatic brain injury, prematurity, extracorporeal membrane oxygenation, and cardiac arrest.

### Procedure

Due to the cumbersome nature of the data collection, patients were included only on weekdays and outside of night shifts. Patients' eligibility was assessed each morning at the medical examination. When a patient met the inclusion criteria, one investigator was dedicated solely to the procedure and present in the room at the time of norepinephrine's initiation (which defined the T_0_). All the investigators were pediatric intensivists trained in advanced ultrasound. All the patients had central line placed before norepinephrine's initiation. Norepinephrine (noradrénaline, Renaudin™) was infused solely via central venous catheter. The timing of initiation, initial dosing, and subsequent changes were left to the physician's decision. The need for fluid resuscitation before norepinephrine initiation was also left to the physician's discretion according to local practices and current guidelines (20). Subsequent volume expansions were collected at the end of data collection to limit the impact of factors likely to influence cerebral hemodynamics, sedative drugs' dosing was maintained unchanged during recordings. Venous or exhaled carbon dioxide (CO_2_) concentrations were also collected at T_0_ and at the end of the data collection, when available. Because of the observational nature of the study, no additional blood sample was collected during the procedure.

### Measurements and Definitions

Systemic (invasive or non-invasive arterial blood pressure, heart rate (HR) and cardiac output) and cerebral hemodynamics (perfusion and oxygenation) were assessed by the same investigator first at T_0_, then at the steady-state. Our team previously assessed the delay and stability of central venous administration of norepinephrine in children (23): Herein we defined the steady-state (T_ss_) as the dosing achieved at least 30 min following the start of treatment, or 10 min from the last dosing modification. Ultrasonography was performed using a VIVID S5^TM^ (GE Healthcare) with a 3MHz probe. For each measure, two windows acquisitions were performed. In case of a difference of more than 20% between the 2 measures, a third one was performed and the mean was recorded. Transthoracic cardiac ultrasound was used to estimate cardiac output (CO). CO was derived from the stroke volume (SV) measured at the apical 5-chamber window, the left ventricular outflow tract (LVOT) diameter, the time velocity integral (TVI), and the heart rate (HR). CO was then calculated as CO=TVI^*^HR^*^ π [LVOT diameter/2]^2^ and indexed to body surface area in order to obtain cardiac index (CI).

Cerebral perfusion was estimated using TCD performed at both trans-temporal windows to assess velocities on the first proximal (M1) segment of middle cerebral arteries (MCA). We measured systolic (Vs), diastolic (Vd), and mean (Vm) velocities, and estimated pulsatility index (PI) as PI=[Vs-Vd)]/Vm, and resistance Index (IR) as RI=[Vs-Vd]/Vs. An increase of Vm and/or a decrease of PI between T_0_ and T_ss_ defined improved cerebral perfusion, whereas the opposite defined altered cerebral perfusion (14).

Cerebral oxygenation was estimated using a 2-wavelength (730–810 nm) cerebral NIRS oximeter (INVOS 5100C^®^, Medtronics, USA). Two sensors were placed on both sides of the patient's forehead to record cerebral tissue oxygen saturation (rScO_2_). Significant cerebral oxygenation variations (ΔrScO_2_) were defined as changes of the rScO_2_ absolute value of more than 20% between T_0_ and T_SS_ (24). We also calculated the cerebral fractional tissue oxygen extraction (cFTOE) as cFTOE = (SpO_2_-rScO_2_)/SpO_2_ (24– 26).

### Statistics

Results are presented with their medians and Interquartile Range (IQR) for continuous variables, and numbers and percentages for categorical variables. To assess the effect of norepinephrine for each patient, we compared the collected parameters between T_0_ and T_ss_ using paired *t*-test or Wilcoxon rank sum test depending on the variables' normality on histograms and Shapiro-Walk tests. A *p*-value < 0.05 defined statistical significance. Data were analyzed using R programming software.

## Results

### Patients' Characteristics

Fourteen children (median [IQR] age of 3.5 [1; 13.5] years) received norepinephrine and were included in this study. Indications for norepinephrine were septic shock (*n* = 12), hemorrhagic shock (*n* = 1) or vasodilatory shock during acute respiratory distress syndrome (*n* = 1). No patient received fluid expansion between T_0_ and T_SS_. Norepinephrine dosing was 0.2 [0.1; 0.32] μg/kg/min at T_ss_. T_SS_ was achieved without modification of norepinephrine's dosing after 30 min for 11 patients. For the 3 left patients, norepinephrine's dosing needed to be increased once. T_SS_ was achieved, respectively, at 40, 45, and 100 min after norepinephrine's initiation. Details are described in Supplementary Table 4. The patients' baseline characteristics are described in Table 1. Twelve patients were hypotensive at the beginning of norepinephrine (mean arterial pressure of 49 [42; 54] mmHg and systolic arterial pressure of 77 [52; 90] mmHg). Heart rate was 117 beats/min [108; 148]. Patients had various systemic hemodynamic profiles with low (*n* = 5), normal (*n* = 5) or high (*n* = 4) CI (Table 1). Individual data are available in Supplementary Table 1.

**Table 1:** Patients baseline characteristics.

	**Patients (*n* = 14) *n* (%) or Median [IQR]**
Age	3.5 [1; 13.5]
**Type of shock**
Septic shock	12 (86)
Hemorragic shock	1 (7)
Vasodilatory shock	1 (7)
PELOD-2	7 [6.8; 8.8]^a^
Lactate (mmol/L)	2.4 [1.3; 3.5]^b^
**Systemic hemodynamic profile**
Clinical signs of inadequate tissue perfusion^c^	14 (100)
Systemic hypotension^d^	12 (86)
**Cardiac index**
<3 L/min/m^2^	5 (36)
>5 L/min/m^2^	4 (27)
Normal cardiac index	5 (36)
**Respiratory characteristics**
Invasive ventilation	12 (85)
FiO_2_ (%)	31 [25; 60]
CO_2_ (mmHg)^e^	43 [38; 51]^a^
SpO_2_ > 92%	14 (100)
Temperature (°C)	37.2 [36.2; 37.8]

*Data are presented with their median [IQR] for continuous variables and numbers (%) for categorical variables*.

a *Missing data for 2 patients*.

b *Missing data for one patient*.

c *Inadequate tissue perfusion was defined as oliguria <0.5 mL/kg/h and/or inadequate cutaneous perfusion (including prolonged capillary refill time) and/or altered mental status*.

d *Hypotension was defined according to systolic and mean blood pressure depending on the patient's age*.

e *CO_2_ was measured by means of venous sample or expired capnography, when available*.

### Systemic and Cerebral Hemodynamics Effects of Norepinephrine for the Entire Group

**Figure 1:**
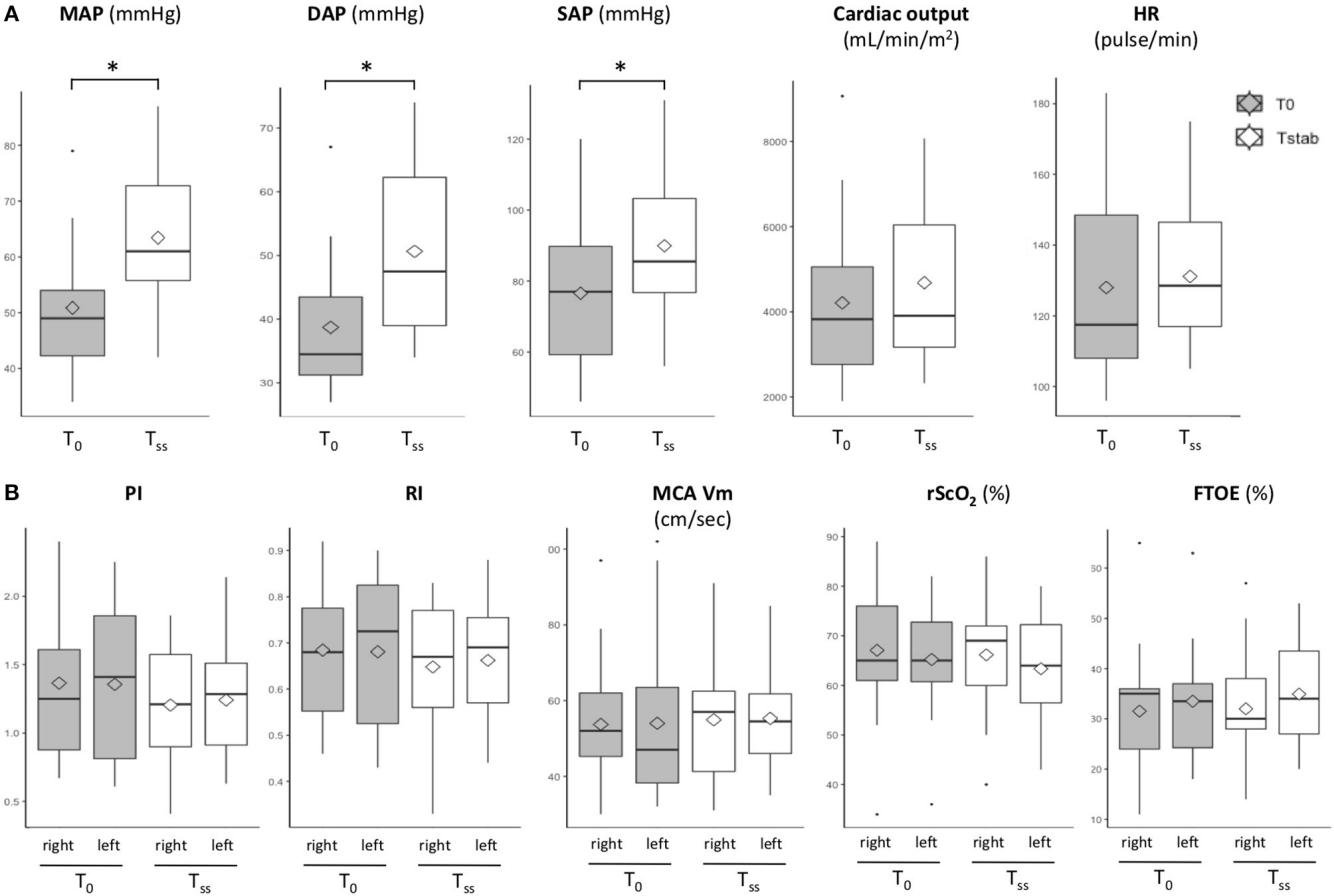
Evolution of **(A)** systemic and **(B)** cerebral hemodynamics during norepinephrine infusion. The central bars and diamonds represent, respectively, the medians and means; the lower and upper ends of the box represent, respectively, the 1st and 3rd quartiles; the T-bars represent the 10th and 90th percentiles; circles are outliers; *p < 0.01. DAP, Diastolic Arterial Pressure; cFTOE, cerebral Fractional Tissue Oxygen Extraction; HR, Heart Rate; MAP, Mean Arterial Pressure; MCA, Middle Cerebral Artery; PI, Pulsatility Index; RI, Resistance Index; SAP, Systolic Arterial Pressure; T_0_, beginning of norepinephrine infusion; T_ss_, steady-state. Median [IQR] cardiac index was 3.8 [2.8; 5.1] mL/min/m_2_ at T_0_ and 3.9 [3.2; 6.0] mL/min/m_2_ at T_SS_. Median [IQR] SAP was 77 [59; 90] mmHg at T_0_ and 86 [77; 103] at T_SS_. Median [IQR] DAP 35 [31; 44] mmHg at T_0_ and 48 [39; 62] mmHg at T_SS_. Median [IQR] MAP was 49 [42; 54] mmHgat T_0_ and 61 [56; 73] mmHg at T_SS_. Median [IQR] HR was 116 [108; 148] bpm at T_0_ and 128 [117; 146] at T_SS_. Median [IQR] right/left PI was 1.4 [0.8; 1.9]/ 1.3 [0.9; 1.6] at T_0_ and 1.3 [0.9; 1.5]/ 1.2 [0.9; 1.6]at T_SS_. Median [IQR] right/left mean MCA velocity was 47 [38; 64]/ 52 [45; 62] cm/sec at T_0_ and 54 [46; 62]/ 57 [41; 62] cm/sec at T_SS_. Median [IQR] right/left rScO_2_ was 65 [61; 73]/ 65 [61; 76] % at T_0_ and 64 [57; 72]/ 69 [60; 72] % at T_SS_. Median [IQR] cFTOE was 34 [24; 37]/ 35 [24; 36] % at T_0_ and 34 [27; 44]/ 30 [28; 38] at T_SS_.

Norepinephrine significantly increased arterial blood pressure (median [IQR] MAP of 61 [56; 73] mmHg at T_ss_ vs. 49 [42; 54] mmHg at T_0_, *p* = 1.10^−3^) without significant change of CI and HR (Figure 1A) for the entire population.

Median [IQR] right/left MCA velocities at T_0_ were, respectively, of 90 [76; 114]/ 98 [80; 115], 24 [18; 39]/ 27 [19; 40], and 47 [38; 64]/ 52 [45; 62] cm/sec for systolic, diastolic, and mean velocities. Median [IQR] right/left PI and RI were 1.4 [0.8; 1.9]/ 1.3 [0.9; 1.6] and 0.7 [0.5; 0.8]/ 0.7 [0.6; 0.8] at T_0._ Cerebral perfusion did not significantly change between T_0_ and T_ss_ (Figure 1B).

Median [IQR] right/left rScO2 and cFTOE were 65 [61; 73]/ 65 [61; 76] % and 34 [24; 37]/ 35 [24; 36] % at T_0_. Cerebral oxygenation did not significantly change between T_0_ and T_SS_ (Figure 1B).

Among the included patients, three had pre-existing neurological abnormalities: two patients had infectious encephalitis without meningitis, and one patient was managed for hyperammoniemic coma in the setting of an organic aciduria (Supplementary Table 1). To avoid for potential neurological confounders, we performed secondary analyses without these patients: cerebral perfusion and oxygenation did not significantly change in the entire population (Supplementary Table 2).

### Patients' Individual Effects of Norepinephrine on Cerebral Hemodynamics

While blood pressure increased in all the patients, some individuals observed a moderate variations of the TCD measured CBF velocities, which slightly improved in 7 (median right/left MCA Vm of 38[33; 45]/38[33; 45] cmH_2_O/s at T_0_ and 50[35; 56]/57[47; 63] at T_SS_; median right/left PI of 1.98[1; 2.1]/1.63[1.5; 2.3] at T_0_ and 1.39[0.9; 1.5]/1.44[1.1; 1.6]), decreased in 2 (median right/left MCA Vm of 74[59; 88]/74[59; 88] cmH_2_O/s at T_0_ and 48[43; 53]/45[38; 53] at T_SS_; median right/left PI of 1.1[0.9; 1.3]/1.6[1.3; 1.9] at T_0_ and 1[0.9; 1.1]/1.32[1.1; 1.6] at T_SS_), and remained unchanged in 5.

Addressing cerebral oxygenation, only one patient (patient 1) had rScO_2_ <50% at T_0_ and T_SS_. No patient experienced a ΔrScO_2_>20% during norepinephrine infusion (Supplementary Table 3).

**Figure 2:**
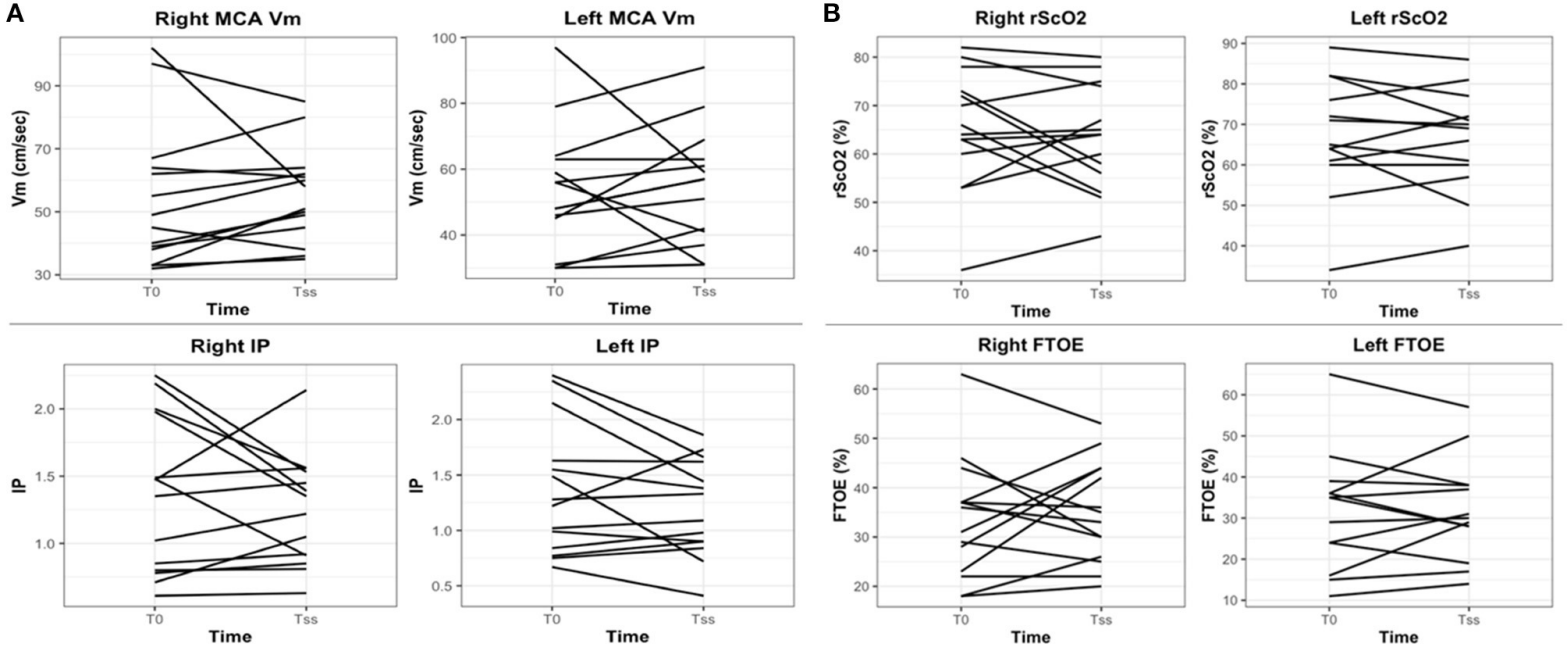
Individual effects of norepinephrine infusion on **(A)** cerebral perfusion and **(B)** cerebral oxygenation. Each line represents one patient. cFTOE, cerebral Fractional Tissue Oxygen Extraction; MCA Vm, Middle Cerebral Artery mean velocity; PI, Pulsatility Index; T_0_, beginning of norepinephrine infusion; T_ss_: steady-state.

The evolution of cerebral hemodynamics during norepinephrine infusion seemed to be highly variable between patients regarding both perfusion and oxygenation (Figure 2). This pattern of evolution seemed to be similar in the 3 patients with previous neurologic injuries (Supplementary Table 3).

## Discussion

At the best of our knowledge, this study is the first to investigate the cerebral hemodynamic effects of norepinephrine in children with shock. Low-dosing norepinephrine induced a homogeneous and significant increase in arterial blood pressure, but had variable effects on cerebral perfusion and oxygenation, suggesting uncoupling between systemic and cerebral hemodynamics. The restoration of systemic macrocirculation does not always reverse tissue hypoxia. Conversely, endothelial dysfunction leading to cellular hypoxia might be present long before the alteration of macrocirculation. In critically ill children, this loss of hemodynamic coherence (28) is common, and associated with a poor outcome (2).

As expected, our patients experienced an increase in blood pressure during norepinephrine infusion. In contrast, CI and HR remained relatively stable given the modest norepinephrine related ß1-inotropic and chronotropic effects. Norepinephrine effects on CO were however variable between individuals. Norepinephrine's cardiac effects might vary depending on the cardiac preload [venous return and his determinants such as the mean systemic filling pressure and the vascular resistance (27, 29)], cardiac afterload, contractility (30), and coronary perfusion (30, 31). In adults, norepinephrine increases heart contractility by several ways including the direct ß1-inotropic effects and the increase in diastolic arterial pressure which improve coronary perfusion. Myocardial hypoxia is less frequent in children, who may be less susceptible to coronary perfusion than adults, and the effect on cardiac output of norepinephrine has been less studied in this population. We presume that in our study some of the patients had preserved stroke volume at baseline, which limited the benefits of norepinephrine on their CO.

Despite norepinephrine induced a homogeneous increase in MAP, cerebral perfusion assessed by TCD did not improve in the entire population. However, the patients' time course evolution suggests that the effect of norepinephrine on cerebral perfusion may differ between individuals (Figure 2, Supplementary Table 4). The between-subjects variability of norepinephrine effects on macrocirculation has already been shown in critically ill children and is related with age, weight, and severity of the disease (13), but has never been demonstrated regarding cerebral perfusion. Cerebral perfusion reflected by cerebral blood flow (CBF) might be easily assessed in children using TCD. In this population, CBF is estimated by means of MCA velocities (15, 16), but the normal ranges for MCA velocities differ depending on age, sex and sedation (14). Baseline flows should be interpreted as standard deviations from normative values. CBF represents the amount of blood that reaches the brain, thus it should depend partly on macrocirculation, notably the stroke volume and the CO. Yet, in our study, MCA velocities did not seem to evolve in the same way than CO for each patient (Supplementary Table 3). CBF indeed depends on multiple factors including also the cerebrovascular reactivity. The preserved CBF suggested by maintained MCA Vm might be explained by a lesser cerebral vasoconstriction, due to the lower density of α-adrenergic receptors on cerebral cortex than on kidneys and skin (32), and/or by a preserved cerebral autoregulation. Static and dynamic cerebral autoregulation assessed by TCD is maintained in adults with sepsis (7). Norepinephrine, by impacting vascular resistances, might change the ability of brain to maintain stable CBF with autoregulation. In the youngest children (i.e., neonates and infants) who have impaired CAR with a shorter autoregulation plateau, this phenomenon might be more important and remains unknown. Our study design lacked continuous acquisitions of CBF to properly assess cerebral autoregulation. We believe that cerebral autoregulation might be preserved given that our patients were managed for a moderate shock, this, corroborated by the modest elevation of blood lactate and PELOD2 score. We have to be cautious when interpreting this result because we did not perform continuous monitoring of blood carbon dioxide (CO_2_). It cannot be ruled out that some patients may have experienced significant CO_2_ variations during norepinephrine infusion, which could impact cerebral vasoreactivity. The neurological benefit of treatments targeting systemic hemodynamics in patients with preserved CAR is unknown. In this population, acute brain dysfunction may be the consequence of other pathological mechanisms (such as blood brain barrier dysfunction, microglial activation, or microcirculation dysfunction), which may not be impacted by norepinephrine.

CBF and vasoreactivity are also influenced by the brain metabolic demand, a phenomenon termed as neurovascular coupling (33). To better investigate the effects of norepinephrine on neurovascular coupling in children with shock, and further assess the link between systemic and cerebral hemodynamics, we sought to approximate cerebral oxygen consumption using NIRS. NIRS technology allow a simple, continuous, and non-invasive calculation of rScO_2_ and cFTOE (34). Contrary to MCA velocities values, the definition of cerebral oxygenation impairment using NIRS is similar among children of all ages (25). In the present study, all the patients except one had normal cerebral oxygenation (rScO_2_) at T_0_ (Supplementary Table 3). Cerebral oxygen extraction estimated by cFTOE remained constant at T_ss_, without variability between subjects. We might assume that patients had stable cerebral oxygen consumption since temperature and sedatives were unchanged during the measurement period. Therefore, norepinephrine introduction did not seem to influence cerebral oxygenation in case of a less severe shock. Cerebral oxygenation and perfusion might evolve in different ways. Mismatch between an improved cerebral perfusion and a stable oxygenation induced by norepinephrine has been previously found in piglets (35) and in adults with sepsis (9). RScO_2_ is sometimes described as a surrogate of CBF (25) but should be used as a complementary monitoring tool with TCD to estimate cerebral FTOE, and consequently assess the adequacy between cerebral oxygen delivery and consumption.

The main limitation of the present study is the small size of the population and the heterogeneous baseline and clinical conditions that might lead to the between-subjects variability observed in cerebral hemodynamics time course. However, we did not find such variability regarding blood pressure evolution despite the population's heterogeneity. Because our study design was descriptive and exploratory only, we did not perform a formal sample size calculation. We might assume that our study lacked power to demonstrate any significant impact of norepinephrine on cerebral circulation due to this small sample size. Second, the technical limits of the devices that we used allow only an indirect estimation of cerebral hemodynamics. NIRS is presumed to assess chromophores (Hemoglobin and Deoxyhemoglobin) concentrations at a level of 2 to 4 cm from its cutaneous sensor, where the microvessels stand. Thus, measured rScO_2_ reflects cerebral oxymetry solely of the cortical frontal (or temporo-parietal) region, and might depend on age, weight, and the presence of edema. Normal values of FTOE have not been validated in all pediatric age range outside the neonatal period. To a lesser extent, TCD might be misinterpreted in case of poor acquisition windows. Finally, the absence of patients with higher norepinephrine dosing prevents us from any conclusion about its effects in severe shocks. It is conceivable that greater amplitude of norepinephrine-induced changes in MAP could have resulted in a larger, and therefore more significant, impact on cerebral perfusion and oxygenation. We also admit that some patients probably had suitable or nearly normal cerebral flow and oxygenation at T_0._ If cerebral hemodynamics were not impaired at baseline due to sufficient compensatory mechanisms, this might explain the absence of norepinephrine's significant effect between T_0_ and T_SS_ regarding cerebral circulation.

Despite these limitations, the rigorous assessment of both cerebral and systemic hemodynamics changes in real time allowed us to describe for the first time the variable effects of low-dosing norepinephrine on cerebral hemodynamics, which might be unpredictable regarding the evolution of systemic hemodynamics. Physicians should be aware of this potential between-subjects variability to target therapy according to the patient's systemic, but also cerebral hemodynamics. This reinforces the need for an individualized monitoring in critically ill children. This original study emphasizes the importance of monitoring regional and cerebral circulation during pediatric shock. We believe that these preliminary results should lead to further the investigations on the cerebral effects of vasoactive and inotropic drugs in critically ill children.

This study has been promoted by Assistance Publique-Hôpitaux de Paris (AP-HP).

## Data Availability Statement

The original contributions presented in the study are included in the article/Supplementary Material, further inquiries can be directed to the corresponding author/s.

## Ethics Statement

The studies involving human participants were reviewed and approved by French Institutional Review Board South-East IV (CPP SO IV), ref 2018-A01392-53. Written informed consent from the participants' legal guardian/next of kin was not required to participate in this study in accordance with the national legislation and the institutional requirements.

## Author Contributions

MV-C, SR, and MO conceptualized and designed the study. MV-C, JC, MG, and P-LL performed the ultrasound exams and collected the data in ICU. MV-C performed analyses and interpretation of data and drafted the first manuscript. MO supervised the analysis and writing of the manuscript. All authors revised the article critically and approved the final version to be published.

## Conflict of Interest

The authors declare that the research was conducted in the absence of any commercial or financial relationships that could be construed as a potential conflict of interest.

## Publisher's Note

All claims expressed in this article are solely those of the authors and do not necessarily represent those of their affiliated organizations, or those of the publisher, the editors and the reviewers. Any product that may be evaluated in this article, or claim that may be made by its manufacturer, is not guaranteed or endorsed by the publisher.
