# Supplementary material for: Low Dosing Norepinephrine Effects on Cerebral Oxygenation and Perfusion During Pediatric Shock
Source: Front Pediatr. 2022 Jul 6;10:898444. doi: 10.3389/fped.2022.898444 (PMC9298794; doi:10.3389/fped.2022.898444)
Supplement: Supplementary file 1 [file Data_Sheet_1.DOCX]

**SUPPLEMENTAL DIGITAL CONTENT**

**Online supplemental table (S1) :** Patients’ individual characteristics at the inclusion

| ID | Age (y) | Indication | Comorbidity | Previous drug | MV | Neurological features | Lactate (mmol/L) | EtCO_2_/PvCO_2_ (mmHg) | T (°C) | Hb  (g/L) | Posology T_ss_(γg/kg/min) |
| --- | --- | --- | --- | --- | --- | --- | --- | --- | --- | --- | --- |
| 1 | 14 | Vasodilatory ARDS | Chronic granulomatous disease | None | Yes | None | 3.7 | 38 | 37.3 | 7.1 | 0.1 |
| 2 | 3 | Septic + hemorragic shock | Congenital cardiopathy | Epinephrine | Yes | None | 1.6 | 56 | 35.5 | 12.1 | 0.1 |
| 3 | 0 | Septic shock | Congenital lactic acidosis | None | Yes | Metabolic coma | 3.5 | 51 | 36.4 | 11 | 0.33 |
| 4 | 12 | Septic shock | Bone marrow transplant | None | Yes | Encephalitis | 8.4 | 38 | 35 | 8.4 | 0.36 |
| 5 | 0.1 | Septic shock | Supraventricular tachycardia | None | Yes | None | 3.1 | 67 | 36 | 10.8 | 0.09 |
| 6 | 4 | Septic shock | None | None | No | None | 1.3 | 51 | 37.9 | NA | 0.1 |
| 7 | 15 | Septic toxinic shock | None | None | Yes | None | 2.4 | NA | 37.5 | 10 | 0.1 |
| 8 | 0.4 | Septic shock | Congenital cardiopathy | None | Yes | None | 1.9 | 34 | 33 | 8.1 | 0.3 |
| 9 | 11 | Septic shock | Bone marrow transplant | None | Yes | None | 0.8 | 51 | 38.2 | 7.5 | 0.35 |
| 10 | 16 | Septic shock | Heart transplant rejection | Milrinone | No | None | 0.8 | 30 | 36.8 | 9.2 | 0.2 |
| 11 | 2 | Septic shock | None | None | Yes | None | 0.8 | 40 | 37.4 | 8.7 | 0.1 |
| 12 | 0.8 | Septic shock | None | None | Yes | Encephalitis | NA | NA | 37.3 | 9 | 0.2 |
| 13 | 1.7 | Septic shock | Type II glycogenosis | None | Yes | Isolated neuromuscular defect | 4.5 | 43 | 39 | NA | 0.2 |
| 14 | 16 | Septic shock | Auto-immune disorder | None | Yes | None | 2.5 | 46 | 39.7 | 9.6 | 0.45 |

ARDS: Acute Respiratory Distress Syndrome; ID: patient number; y: year; MV: Invasive mechanical ventilation; Hb: Haemoglobin; NA: non available; EtCO_2_/PvCO_2_: expired capnography or venous partial pressure of carbon dioxide

**Online supplemental table (S2):** secondary analysis of cerebral hemodynamics evolution during norepinephrine infusion without the 3 brain-injured patients (n=11).

|  | T_0_  Median [IQR] | T_ss_  Median [IQR] | p |
| --- | --- | --- | --- |
| rScO_2_ (%) |  |  |  |
| Right | 66 [56 ; 72] | 64 [57 ; 70] | 0,66 |
| Left ^a^ | 67 [60 ; 79] | 69 [61 ; 71] | 0,95 |
| cFTOE (%) |  |  |  |
| Right | 31 [25 ; 40] | 35 [28 ; 43] | 0,78 |
| Left ^a^ | 32 [18 ; 38] | 29 [28 ; 39] | 0,82 |
| MCA systolic velocity (cm/sec) |  |  |  |
| Right | 90 [75 ; 109] | 97 [89 ; 114] | 0,36 |
| Left | 100 [82 ; 112] | 84 [74 ; 114] | 0,92 |
| MCA diastolic velocity (cm/sec) |  |  |  |
| Right | 23 [18 ; 31] | 31 [24 ; 42] | 0,18 |
| Left | 24 [19 ; 33] | 26 [21 ; 45] | 0,19 |
| MCA mean velocity (cm/sec) |  |  |  |
| Right | 45 [38 ; 63] | 51 [47 ; 62] | 0,11 |
| Left | 48 [45 ; 61] | 57 [39 ; 66] | 0,44 |
| Pulsatility Index |  |  |  |
| Right | 1,47 [0,9 ; 1,7] | 1,35 [0,9 ; 1,4] | 0,34 |
| Left | 1,28 [1 ; 1,5] | 1,33 [0,9 ; 1,5] | 0,17 |

Continuous variables are presented in median [IQR]. cFTOE: cerebral Fractional Tissue Oxygen Extraction; MCA: Middle Cerebral Artery; SD: Standard Deviation; T_0_: beginning of norepinephrine infusion; T_ss_: steady-state.

^a^ Missing data for one patient

**Online supplemental table (S3) :** Patients’ individual evolution between T_0_ and T_SS_

| ID | Age (y) | MAP (mmHg) | | CI (L/min/m2) | | rScO_2_ (%)  Right/Left | | ∆rScO_2_ (%) Right/Left | | FTOE (%)  Right/Left | | MCA Vm (cm/sec)  Right/Left | | Age normal values for MCA Vm (from (1)) | PI  Right/Left | | RI  Right/Left | |
| --- | --- | --- | --- | --- | --- | --- | --- | --- | --- | --- | --- | --- | --- | --- | --- | --- | --- | --- |
|  |  | T_0_ | T_ss_ | T_0_ | T_ss_ | T_0_ | T_ss_ | |  | T_0_ | T_ss_ | T_0_ | T_ss_ | Mean(sd) | T_0_ | T_ss_ | T_0_ | T_ss_ |
| 1 | 14 | 49 | 86 | 4.0 | 5.3 | 36/34 | 43/40 | | 7/6 | 63/65 | 53/57 | 49/48 | 60/57 | 61(21)/ 58(14)* | 1.48/1.49 | 0.91/0.72 | 0.74/0.75 | 0.57/0.49 |
| 2 | 3 | 79 | 78 | 3.8 | 6.7 | 82/89 | 80/86 | | -2/-3 | 18/11 | 20/14 | 39/56 | 45/41 | 75 (25) | 0.85/1.28 | 0.92/1.33 | 0.54/0.69 | 0.57/0.71 |
| 3 | 0 | 40 | 42 | 2.8 | 2.9 | 78/76 | 78/81 | | 0/5 | 22/24 | 22/19 | 33/30 | 35/42 | 38 (14) | 2.00/2.4 | 1.56/1.86 | 0.86/0.92 | 0.76/0.83 |
| 4 | 12 | 61 | 63 | 3.1 | 3.6 | 63/64 | 51/50 | | -12/-14 | 37/36 | 49/50 | 102/97 | 58/59 | 61(21)/ 58(14)* | 0.71/0.77 | 1.05/0.90 | 0.48/0.51 | 0.62/0.56 |
| 5 | 0.1 | 40 | 58 | 3.9 | 3.9 | 72/NA | 56/NA | | -16/NA | 28/NA | 44/NA | 32/31 | 36/37 | 38 (14) | 1.98/1.55 | 1.35/1.38 | 0.85/0.76 | 0.71/0.72 |
| 6 | 4 | 46 | 76 | 3.9 | 3.9 | 80/82 | 74/71 | | -6/-11 | 18/16 | 26/29 | 64/63 | 61/63 | 75 (25) | 0.61/0.67 | 0.63/0.41 | 0.43/0.46 | 0.44/0.33 |
| 7 | 15 | 41 | 61 | 2.4 | 2.3 | 70/64 | 75/72 | | 5/8 | 29/35 | 25/28 | 62/30 | 64/31 | 61(21)/ 58(14)* | 1.35/1.02 | 1.45/1.09 | 0.71/0.61 | 0.74/0.63 |
| 8 | 0.4 | 34 | 50 | 5.4 | 5.4 | 66/82 | 52/77 | | -14/-5 | 31/15 | 44/17 | 40/46 | 49/51 | 38(14) | 2.25/2.35 | 1.53/1.66 | 0.9/0.92 | 0.76/0.79 |
| 9 | 11 | 54 | 62 | 9.1 | 7.5 | 53/60 | 67/60 | | 14/0 | 46/39 | 30/38 | 67/64 | 80/79 | 61(21)/ 58(14)* | 2.19/2.15 | 1.39/1.44 | 0.89/0.88 | 0.72/0.74 |
| 10 | 16 | 67 | 87 | 2.0 | 3.0 | 53/61 | 60/66 | | 7/5 | 44/36 | 35/28 | 45/59 | 38/31 | 61(21)/ 58(14)* | 1.47/1.22 | 2.14/1.73 | 0.74/0.67 | 0.88/0.80 |
| 11 | 2 | 51 | 55 | 6.9 | 6.2 | 73/72 | 58/69 | | -15/-3 | 23/24 | 42/31 | 97/79 | 85/91 | 66(21) | 0.78/0.84 | 0.85/0.98 | 0.51/0.54 | 0.54/0.59 |
| 12 | 0.8 | 47 | 50 | 2.8 | 3.7 | 64/65 | 65/61 | | 1/-4 | 36/35 | 33/37 | 55/56 | 62/61 | 58(15) | 0.80/0.75 | 0.81/0.84 | 0.52/0.5 | 0.53/0.54 |
| 13 | 1.7 | 49 | 59 | 1.9 | 3.0 | 63/71 | 64/70 | | 1/-1 | 37/29 | 36/30 | 38/48 | 50/57 | 66 (21) | 1.49/1.63 | 1.56/1.62 | 0.75/0.78 | 0.76/0.78 |
| 14 | 16 | 54 | 61 | 7.1 | 8.1 | 60/52 | 64/57 | | 4/5 | 37/45 | 30/38 | 33/45 | 51/69 | 61(21)/ 58(14)* | 1.02/0.99 | 1.22/0.90 | 0.61/0.59 | 0.67/0.56 |

Cerebral perfusion increased in patients 1, 3, 5, 8, 9, 13 and 14 after norepinephrine (attested by an increase of MCA Vm with or without decrease of PI), decreased in patients 4 and 10 (attested by a decrease of MCA Vm associated with an increase in PI), and was unchanged in others.

Cerebral oxygenation was stable in all the patients (∆rScO_2_ <20% between T_0_ and T_SS_). Only the patient 1 had a low baseline rScO_2_ (<50%).

CI: Cardiac Index; FTOE: cerebral Fractional Tissue Oxygen Extraction; ID: patient number; MAP: Mean Arterial Pressure; MCA: Middle Cerebral Artery; NA: non available; PI: Pulastility Index; RI: Resistance Index; sd: standard deviation; T_0_: beginning of norepinephrine infusion; T_ss_: steady-state; Vs : Systolic Velocity; Vd: Diastolic Velocity; Vm: Mean Velocity.

Patients with previous neurological injury are underlined in grey

*First value is for male, second value is for female1. O’Brien NF. Reference values for cerebral blood flow velocities in critically ill, sedated children. Childs Nerv Syst. dec 2015;31(12):2269-76.

**Online supplement figure (S4):** Time-course evolution between T_0_ and T_SS_ for the 3 patients who had more than one norepinephrine titration


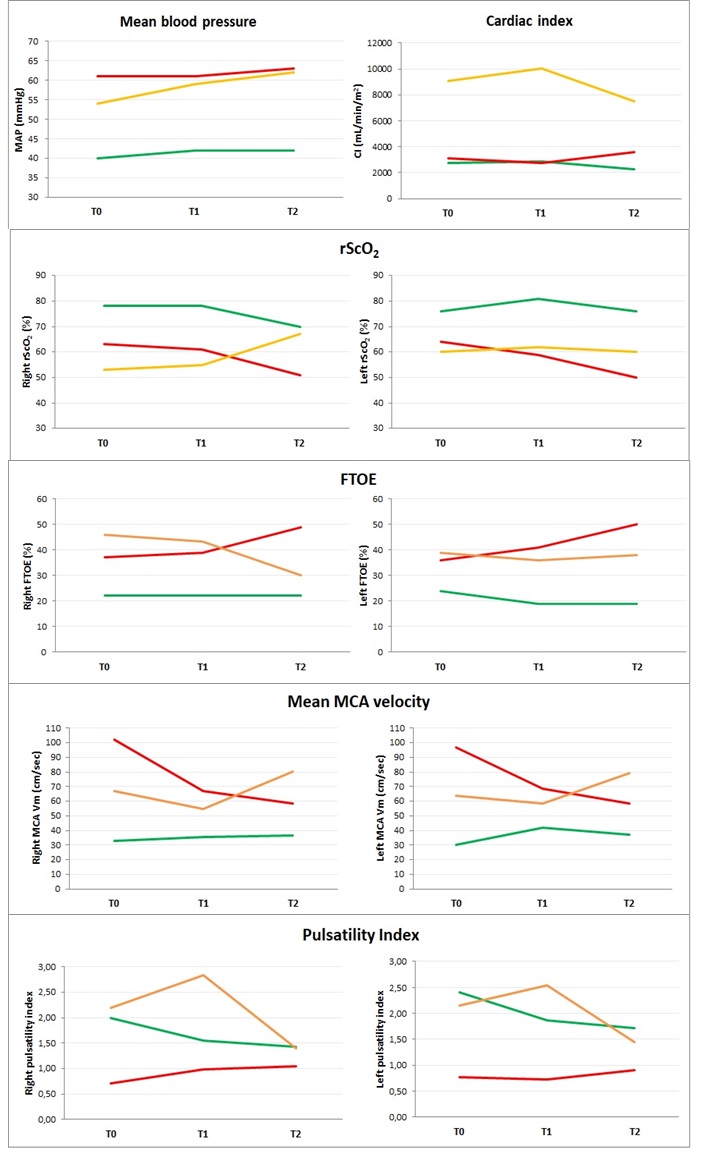


Each line represents one patient. T0 represents the first measurement at the time of norepinephrine initiation. T1 represents the intermediate timepoint during norepinephrine titration to achieve clinical targets (restoration of the mean arterial pressure). T2 represents the steady-state (T_ss_) in these 3 patients, which was defined as the dosing achieved at least 30 minutes following the start of treatment, or 10 minutes from the last dosing modification. T2 was achieved respectively after 40 minutes (green line), 45 minutes (orange line), and 100 minutes (red line).

MAP: Mean arterial pressure; CI: Cardiac index; MCA: Mean cerebral artery; Vm: mean velocity; FTOE: Fractional tissue oxygen extraction.
